# Blood biomarkers role in acute ischemic stroke patients: higher is worse or better?

**DOI:** 10.1186/1742-4933-9-22

**Published:** 2012-10-31

**Authors:** Aliaksei Kisialiou, Giordana Pelone, Albino Carrizzo, Giovanni Grillea, Valentina Trimarco, Marina Marino, Michelangelo Bartolo, Alessandro Marco De Nunzio, Rodolfo Grella, Alessandro Landolfi, Annibale Puca, Claudio Colonnese, Carmine Vecchione

**Affiliations:** 1 Clinical Epidemiology & Biostatistics Unit, IRCCS INM Neuromed, Pozzilli (IS), Italy; 2 Stroke Unit, IRCCS INM Neuromed, Pozzilli (IS), Italy; 3 Vascular Physiopathology Unit, IRCCS INM Neuromed, Pozzilli (IS), Italy; 4 Diagnostical & Therapeutical NeuroRadiology Unit, IRCCS INM Neuromed, Pozzilli (IS), Italy; 5 Department of Neuroscience, University of Naples Federico II, Naples (NA), Italy; 6 Department of Clinical Medicine, Cardiovascular and Immunological Sciences, University of Naples Federico II, Naples (NA), Italy; 7 NeuroRehabilitation Unit, IRCCS INM Neuromed, Pozzilli (IS), Italy; 8 AngioCardioNeurology Unit, IRCCS INM Neuromed, Pozzilli (IS), Italy; 9 Cardiovascular Research Unit, IRCCS Multimedica, Milano, Italy; 10 Department of Medicine and Surgery, University of Salerno, Salerno, Italy

**Keywords:** Biomarkers, Acute stroke, Lesion size, Neuroimaging

## Abstract

**Background:**

Thrombolytic therapy (TT) for acute ischemic stroke (AIS) can provoke bleeding’s complication depending on the ischemic lesion (IL) dimension. Inflammation involved in the setting of acute ischaemic stroke, is associated with infarct size. We aimed to study the independent correlation and association between clinical panel of routinely identified biomarkers, including inflammatory parameters, and cerebral IL dimension and site.

**Results:**

We evaluated eleven biomarkers in 105 unrelated patients during their hospitalization after acute stroke event. Our data indicate a significant association of: a) confluent IL size with 4th quartile of Erythrocyte Sedimentation Rate (ESR) (OR = 5.250; 95% CI, 1.002 to 27.514) and an independent correlation with sex; b) confluent IL size with 3rd quartile of fibrinogen (OR = 5.5; 95% CI, 1.027 to 29.451); c) confluent IL size with 3rd quartile of platelets (OR= 0.059; 95% CI, 0.003 to 1.175) and independent correlation with sex; d) smaller IL size (OR = 5.25; 95% CI, 1.351 to 20.396) with 3rd quartile of albumin levels and nodular and parenchimal IL size with 2nd (OR = 0.227; 95% CI, 0.053 to 0.981), 3rd (OR = 0.164; 95% CI, 0.038 to 0.711) and 4th (OR = 0.205; 95% CI, 0.048 to 0.870) quartiles albumin levels; e) smaller IL size with 3rd quartile triglycerides (TG) levels (OR = 9; 95% CI, 2.487 to 32.567) and an independent correlation with anterior location. Smaller IL size, anterior AIS turned out to be independently correlated with high serum albumin levels. Finally, high INR and PTT values were associated with worse NIHSS clinical outcomes in contrast to that observed with higher albumin level.

**Conclusions:**

We provide evidence of routine biomarkers levels correlation with acute IL size, independently of age and sex. In addition, we highlight the importance of differentiation of biomarkers normal interval levels for further improvement not only of the clinical decision making but also in post-acute clinical outcome management.

## Background

Stroke remains the third leading cause of death and the leading cause of severe disability in the United States, Europe and portions of Asia in young [1] and elderly people [2-4]. Actually, the successful therapy for acute stroke is thrombolysis and bleeding is often a complication that can also depend on the lesion size [5]. Recent report shows that inflammation in the setting of acute ischaemic stroke (AIS) is associated with infarct size, supporting the hypothesis that inflammation in acute stroke primarily reflects an acute phase response determined by the degree of cerebral injury. Acute inflammation that develops following the initial ischemic episode is a major mechanism by which cells in the penumbra degenerate and the participation of inflammatory factors could be associated to the presence of early neurological deterioration and infarct volume [6].

The definition of the available independent correlates of serum biomarkers with cerebral lesions and sites could further help in the clinical practice for the acute ischemic stroke management reducing complications following acute treatment [7]. Despite it, the biomarkers serum levels were hypothesized to be independently correlated with cerebral lesion sites and size in AIS, but so far there are no human data available for this hypothesis [8]. Our aim was to characterize the clinical biomarkers routine panel correlation and association with cerebral lesion sites and dimensions in AIS patients, evaluating the possible association with clinical outcome.

## Results

Our study population includes 105 patients: 59 (56.19%) males and 46 (43.81%) females. The mean age of patients was 63.3 years (SD ± 14 years, range 46–78 years). 9 patients were excluded because of missing data on biomarkers. Thus, the final study population includes 96 participants. Table 1 reports descriptive statistics for classical cerebrovascular risk factors stratified by gender. In particular, women are a bit older than man and their levels of erythrocyte sedimentation rate (ESR), high density lipoprotein (HDL), glucose, hypertension prevalence and anterior stroke site distribution are increased as compared to men. In contrast, serum triglycerides (TG), partial thromboplastin time (PTT) and low density lipoprotein (LDL) values of women are lower compared to men. There are no significant differences between the sexes for other variables. The relationship between plasma biomarkers levels and AIS cerebral lesion site and lesion dimension revealed as regression coefficients with relative p-values are shown in Table 2.

**Table 1:** AIS variables characteristics in the study population

**Variable name (Units)**	**Total (n = 105)**	N**	**Women**	**N**	**Men**	**N**
	Mean* ± SD		Mean* ± SD		Mean* ± SD	
NIHSS at 7 days	6.47 ± 0.6	105	8.4 ± 0.9	46	5.0 ± 0.7	59
Age (years)	63.3 ± 14	105	67.2 ± 14.8	46	60.2 ± 12.7*	59
Body Mass Index (Kg / m^2^)	25.5 ± 3.7	79	25.2 ± 4.1	20	25.7 ± 3.6	59
Hypertension (%)	55.2	58	69.5	32	44.1*	26
Diabetes mellitus (%)	39.1	41	41.3	19	37.3	22
Previous myocardial infarction (%)	14.3	15	15.2	7	13.6	8
Current smoking (%)	17.1	18	10.8	5	22*	13
SBP (mmHg)	142.9 ± 25.7	105	142.6 ± 24.4	46	143.1 ± 26.8	59
DBP (mmHg)	81.03 ± 13.8	105	79.2 ± 14	46	82.3 ± 13.6	59
MAP (mmHg)	101.7 ± 16.4	105	100.3 ± 16	46	102.6 ± 16.7	59
Heart rate (bpm)	72.8 ± 12.9	105	70 ± 13.6	46	72 ± 12.6	59
AIS site (anterior%)	59.1	62	71.7	33	49.2*	29
Glucose (mg / dL)	130.4 ± 52.4	104	140.7 ± 57.4	46	122.1 ± 47.6*	58
Albumin (g / dL)	3.2 ± 0.6	96	3.2 ± 0.7	42	3.4 ± 0.7	54
TG (mg / dL)	129.4 ± 71.4	103	118.1 ±72.1	46	137.9 ± 71.7*	57
TC (mg / dL)	179.7 ± 43.2	103	179.9±42.7	46	179.6 ± 44.7	57
LDL (mg / dL)	122.9 ± 41.3	102	116.3 ± 37.7	46	127.9 ± 44.6*	56
HDL (mg / dL)	41.70 ± 11.4	102	45.6 ± 10.1	46	38.8 ± 11.9*	56
INR (ratio)	1.15 ± 0.5	105	1.20 ± 0.8	46	1.08 ± 0.1	59
PTT (sec)	30.9 ± 5.7	104	28.4 ± 20.6	46	30.8 ± 4.1	58
Platelets (value x 10.e^3^ / uL)	262.2 ± 92	100	302.8 ± 108.4	46	234.1 ± 71.5*	54
ESR (mm)	21.3 ± 19.8	99	26.8 ± 24.3	45	17.3 ± 16.1*	54
Fibrinogen (mg / dL)	398.1 ± 137.1	98	396.1 ± 177.5	44	390.6 ± 123.8	54

All values are unadjusted means ± standard deviation for continuous trait and percent for non-continous trait.* significantly different from women. **N = number of samples for each biomarker analyzed.

**Table 2:** Independent correlates of principal biomarkers in AIS

**Biomarker**	**ESR (mm)**		**Fibrinogen (mg / dL)**		**Platelets (val. x 10.e**^**3**^**/ uL)**		**Blood albumin (g / dL)**		**Triglycerides (mg / dL)**		**Glucose (mg / dL)**		**HDL (mg / dL)**	
	**b ± S.E.**	**p**	**b ± S.E.**	**p**	**b ± S.E.**	**p**	**b ± S.E.**	**p**	**b ± S.E.**	**p**	**b ± S.E.**	**p**	**b ± S.E.**	**p**
Site							+ 0.48 ± 0.23	0.044	+ 0.63 ± 0.24	0.008*				
Sex	- 0.65 ± 0.24	0.008*			- 0.81 ± 0.23	0.001*							- 0.66 ± 0.23	0.005*
D1					- 0.02 ± 0.01	0.029	- 0.03 ± 0.008	0.002*			+ 0.02 ± 0.008	0.018*		
D2					- 0.68 ± 0.31	0.035	- 0.59 ± 0.25	0.020	+ 0.68 ± 0.25	0.009*				
D3	+ 0.53 ± 0.27	0.054*	+ 0.58 ± 0.27	0.040*			- 0.55 ± 0.27	0.042						
D4							- 0.98 ± 0.47	0.041						

No independent correlates were identified for dependent variables of TC, LDL, INR; * p - value < 0.01; D1 = points (< 1.5 cm], D2 = nodular dimensions [1.5 - 3 cm] & parenchymal dimensions (> 3 cm), D3 = confluent dimensions, D4 = non confluent dimensions.

As anticipated by significant magnitudes and directions between biomarkers and lesion sites revealed by regression coefficients, given the random variance introduced by different levels of biomarkers among AIS patients, further relationship between biomarker’s quartiles: first quartile (Q1) used as reference to second quartile (Q2), third quartile (Q3), forth quartile (Q4) and lesion location and size were assessed.

**Figure 1:**
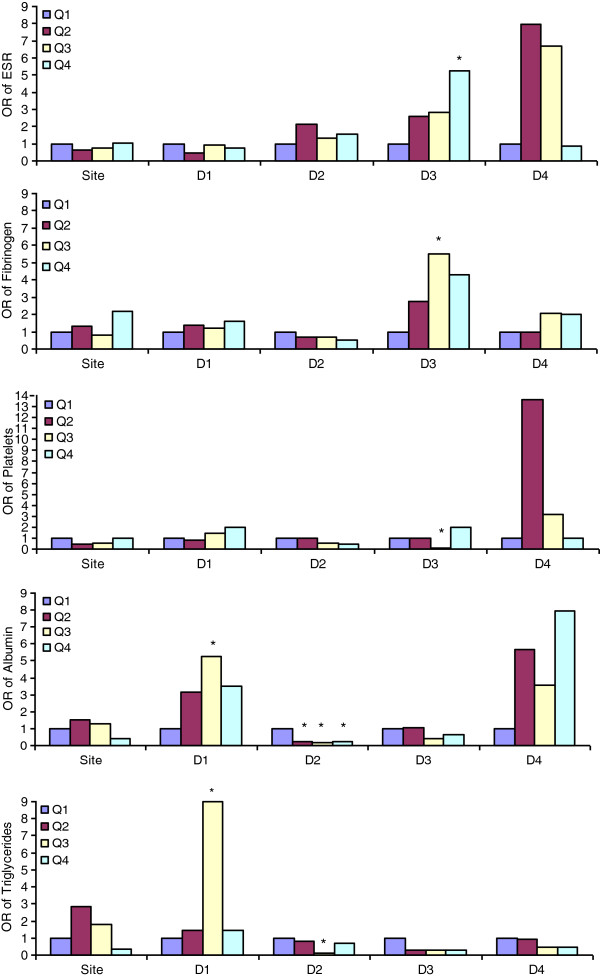
**Biomarkers OR stratified by AIS lesion site and dimension (D1 - D4).** Patients in Q1 were used as a reference in each group. All values were adjusted for age and sex. *Significantly different from Q1.

ESR serum levels were independently correlated with sex (b = + 0.65, S.E. = 0.24, p = 0.008) and D3 (b = + 0.53, S.E. = 0.27, p = 0.0045) lesion (Table 2). In quartile analysis D3 lesion remained associated with Q4 (OR, 5.250; 95% CI, 1.002 - 27.514 ) of ESR (Table 3) (Figure 1).

**Table 3:** Multivariate testing: OR (95% CI) to the first quartile of biomarkers investigated vs stroke lesion site (anterior vs posterior) & stroke lesion dimensions

**Variable (Units) / Quartiles**	**Site**	**D1**	**D2**	**D3**	**D4**
	**(OR; 95% CI)**	**(OR; 95% CI)**	**(OR; 95% CI)**	**(OR; 95% CI)**	**(OR; 95% CI)**
ESR (mm)					
Q1 (< 10) n = 23	1	1	1	1	1
Q2 [10 – 16) n = 30	0.650 (0.212-1.993)	0.473 (0.149-1.501)	2.115 (0.666-6.715)	2.625 (0.478-14.428)	7.981 (0.383-166.240)
Q3 [16 – 30) n = 19	0.758 (0.218-2.632)	0.945 (0.277-3-230)	1.319 (0.380-4.577)	2.800 (0.452-17.318)	6.714 (0.283-159.527)
Q4 [> 30) n = 27	1.040 (0.339-3.190)	0.765 (0.246-2.381)	1.538 (0.488-4.853)	5.250 (1.002-27.514)*	0.855 (0.015-48-430)
Fibrinogen (mg/dL)					
Q1 (< 303) n = 24	1	1	1	1	1
Q2 [303 – 368) n = 25	1.333 (0.415-4.281)	1.366 (0.411-4.539)	0.708 (0.203-2.470)	2.750 (0.479-15.794)	0.958 (0.057-16.243)
Q3 [368 – 462) n = 24	0.824 (0.242-2.797)	1.214 (0.358-4.124)	0.667 (0.190-2.338)	5.500 (1.027-29.451)*	2.091 (0.177-24.734)
Q4 [> 462) n = 25	2.167 (0.682-6.883)	1.619 (0.493-5.319)	0.500 (0.147-1.697)	4.278 (0.789-23.193)	2.000 (0.169-23.623)
Platelets (value x 10.e3/uL)					
Q1 (< 189) n = 25	1	1	1	1	1
Q2 [189 – 256) n = 25	0.434 (0.138-1.371)	0.826 (0.246-2.776)	1.000 (0.291-3.437)	1.000 (0.278-3.598)	13.683 (0.674-277.964)
Q3 [256 – 323) n = 25	0.519 (0.167-1.611)	1.417 (0.444-4.521)	0.583 (0.178-1.906)	0.059 (0.003-1.175)*	3.122 (0.114-85.732)
Q4 [> 323) n = 25	1.000 (0.330-3.033)	1.962 (0.621-6.193)	0.495 (0.153-1.606)	2.032 (0.608-6.797)	1.000 (0.018-56.631)
Blood Albumin (g/dL)					
Q1 (< 2.9) n = 25	1	1	1	1	1
Q2 [2.9 – 3.4) n = 24	1.504 (0.488-4.639)	3.150 (0.815-12.168)	0.227 (0.053-0.981)*	1.059 (0.306-3.658)	5.677 (0.243-132.298)
Q3 [3.4 – 3.8) n = 22	1.273 (0.403-4-19)	5.250 (1.351-20.396)*	0.164 (0.038-0.711)*	0.406 (0.091-1.817)	3.558 (0.129-98.252)
Q4 [> 3.8) n = 25	0.402 (0.102-1.349)	3.500 (0.921-13-307)	0.205 (0.048-0.870)*	0.643 (0.173-2.388)	7.933 (0.366-172.044)
Triglycerides (mg/dL)					
Q1 (< 78) n = 25	1	1	1	1	1
Q2 [78 – 111) n = 26	2.844 (0.913-8.861)	1.474 (0.398-5.452)	0.833 (0.218-3.179)	0.273 (0.072-1.034)	0.958 (0.124-7.383)
Q3 [111 – 162) n = 26	1.778 (0.579-5.457)	9.000 (2.487-32.567)*	0.132 (0.037-0.471)*	0.273 (0.072-1.034)	0.460 (0.039-5.418)
Q4 [> 162) n = 26	0.323 (0.084-1.237)	1.474 (0.398-5.454)	0.679 (0.183-2.510)	0.273 (0.072-1.034)	0.460 (0.039-5.418)

OR = odds ratio; 95% CI = odds ratio confidence interval. ORs are adjusted for age and sex; * D1 = points (< 1.5 cm], D2 = nodular dimensions [1.5 - 3 cm] & parenchymal dimensions (> 3 cm), D3 = confluent dimensions, D4 = non confluent dimensions; * p-value < 0.05 compared with the reference group Q1; ** N = number of samples = 96.

Fibrinogen serum levels were independently correlated with D3 (b = + 0.58, S.E. = 0.27, p = 0.040) lesion (Table 2). Lesion D3 remained associated with Q3 (OR, 5.500; 95% CI, 1.027 - 29.451 p=0.040) of fibrinogen in quartile analysis (Table 3) (Figure 1). Platelets serum levels were independently correlated with sex (b = + 0.81, S.E. = 0.23, p = 0.001) and D1 (b = + 0.02, S.E. = 0.01, p = 0.003), D2 (b = + 0.68, S.E. = 0.31, p = 0.035) lesions (Table 2). In quartile analysis only D3 lesion remained associated with Q3 (OR, 0.059; 95% CI, 0.003 - 1.175 ) of platelets (Table 3) (Figure 1).

Albumin levels were independently correlated with site (b = − 0.48, S.E. = 0.23, p = 0.0436) and D1 (b = − 0.03, S.E. = 0.008, p = 0.002), D2 (b = − 0.59, S.E. = 0.25, p = 0.020), D3 (b = −0.55, S.E. = 0.27, p = 0.042), D4 (b = − 0.98, S.E. = 0.47, p = 0.041) size (Table 2); lesion D1 size remained associated with Q3 (OR, 5.250; 95% CI, 1.351 - 20.396 ) of serum albumin level, whereas lesion D2 remained associated with Q2 (OR, 0.227; 95% CI, 0.053 - 0.981), Q3 (OR, 0.164; 95% CI, 0.038 - 0.711 ) and Q4 (OR, 0.205 ; 95% CI, 0.048 - 0.870) (Table 3) (Figure 1).

TG levels were independently correlated with site (b = + 0.63, S.E. = 0.24, p = 0.008) and D2

(b = + 0.68, S.E. = 0.25, p = 0.009) size (Table 2). Lesion D1 was associated with Q3 (OR, 9.000; 95% CI, 2.487 - 32.567) of TG and lesion D2 size remained associated with Q3 (OR, 0.132; 95% CI, 0.037 - 0.471 ) (Table 3) (Figure 1).

HDL serum levels were independently correlated with sex (b = + 0.66, S.E. = 0.23, p = 0.005) and glucose levels were independently correlated with D1 (b = + 0.02, S.E. = 0.008, p= 0.018) lesion (Table 2).

In quartile analysis, both HDL and glucose as well as PTT, international normalized ratio (INR), LDL and total cholesterol (TC) (data not shown) were not significantly associated with lesion size dimension.

Finally, high INR (NIHSS ≥ 14, p = 0.01) and high PTT (NIHSS ≥ 7, p = 0.001) were associated with worse clinical outcomes, respectively. In contrast, higher albumin serum level was associated with better clinical outcome (NIHSS < 7, p = 0.006) at 7 days.

## Discussion

The use of biomarkers has made a strong impact in understanding of the pathophysiology of stroke and the approach to its treatment [9]. Actually, the only approved pharmacological therapy for acute stroke is thrombolysis which acts mediating recanalization and often induces secondary hemorrhage depending on the IL size [10]. The identification of clinical biomarkers of acute cerebral ischemia would facilitate the use of time-sensitive reperfusion strategies offering a potential role for improved treatment selection and individualization of therapy.

Brain inflammation contribute to the pathophysiology of cerebral injury in acute stroke and it has been reported to be associated with infarct size [11,12]. We show that high ESR value is associated with larger infarct size. ESR values observed soon after AIS may reflect a degree of acute phase response in early phase of AIS and extent of local brain damage [13]. The acute phase response follows tissue injury and contributes to its exacerbation with pro-inflammatory and pro-thrombotic mechanisms inducing erythrocyte aggregation [14]. Thus, ESR is a measure of the acute phase response and its monitoring at the time of admission may serve as a marker for severity after AIS.

In addition to ESR we found even normal-high fibrinogen serum levels associated with larger infarct size. Fibrinogen, previously described as an independent coronary heart disease risk factor is now considered an inflammatory marker modulating the balance between hemostasis and thrombosis, coagulation and fibrosis, protection from infection and extensive inflammation [15]. A plasma fibrinogen measurement is a clinical standard to evaluate bleeding disorder or thrombotic episode. Apart from its pivotal role in thrombogenesis, inflammation, immune responses and atherogenesis, it is also a prominent acute-phase reactant. Transiently elevated plasma fibrinogen levels have been described in acute stroke. Fibrinogen acts also as bridge between adjacent platelets to generate a platelet aggregate [15]. Circulating platelets have been recognized for their importance in modulating recurrent stroke and we show that high-normal platelet serum levels are associated with large infarct dimension. Circulating platelets play an important role in the development, progression, and resolution of stroke, not only depending on their direct effects on endothelium but also by acting as a connection for other cells in vascular system. In particular, following vessel injury platelets interact with damaged endothelium and release factors stored in granules that play a role in recruitment of leukocytes, additional platelets, or other blood cells to the vessel wall [16]. The interaction among these factors is critical for hemostasis, host defense and represents the mechanism used by platelets to induce atherothrombosis and inflammatory events leading to ischemic stroke.

Among the biochemical parameters used routinely we show that normal-high albumin levels characterize AIS patients with minor lesion size whereas low-normal albumin levels were associated with more extended lesion. Experimental studies showed that high-dose or moderate-dose of human albumin therapy, after stroke onset, is highly effective in improving neurological status and in reducing infarction volume and extent of brain swelling [17-19]. Albumin is the most abundant protein found in plasma, functioning as a carrier molecule, maintaining oncotic pressure and acting as a major antioxidant defender in inflammatory process [18]. In addition, serum albumin level is one of the biochemical markers of nutritional status and it has been reported that protein-energy malnutrition after acute stroke is a risk factor for poor outcome worsening the prognosis [20]. These experimental evidences are in agreement with our finding observed in humans showing that low serum levels of albumin are associated with elevated NIHSS score at 7 days, suggesting that high albumin is neuroprotective in ischemic stroke for both lesion gravity and clinical outcome [21,22].

Finally, we found that normal-high levels of TG are associated with smaller infarct size. The role of TG in the risk of ischemic stroke remains controversial. A strong association was found between elevated levels of TG and increased risk of ischemic heart disease [23]. In addition, increased levels of nonfasting TG were correlated with an incidence of ischemic stroke [24]. Nonfasting TG indicate the presence of increased levels of remnants from chylomicrons and very low-density lipoproteins which penetrate the arterial endothelium and may get trapped within the subendothelial space, potentially leading to the development of inflammation in atherosclerosis.

In addition, both high INR and PTT were associated with worse NIHSS outcome, suggesting their neuroprotective effect at low levels in acute stroke patients.

Regarding the independent correlation to lesion site we observed high-normal significant values of albumin and TG serum levels and we were not able to establish the association between quartiles of other biomarkers and lesion site.

## Conclusions

Our results show the association of some inflammatory biomarkers with ischemic lesion size suggesting the contribution of inflammation as a prognostic indicator for the development of clinical complications following cerebral acute events. Actually, there is a substantial interest in the use of biomarkers panel to identify subjects at higher risk for the development of complications following thrombolysis therapy. The present study examines the association between biomarkers, paying attention, in particular, to the hypothesis that different plasma levels of biomarkers assessed at baseline in subjects affected by AIS could be predictive for developement of both size lesions and clinical outcome. These results further confirm our assumption that multivariate analyses of relevant biomarkers are necessary to reduce the risk of inaccurate prognosis. It is important to emphasize that considering biomarker’s normal range still does not allow to exploit their clinical potential in management of AIS. We highlight the importance of differentiation of normal interval levels of serum biomarkers to improve not only clinical decision making but also post acute clinical outcome.

Exploratory studies within clinical trials are necessary before blood markers of cerebral tissue damage can be recommended as surrogate endpoints. In other words it is essential to increase the number of informative markers and to assess their relative contributions to diagnosis, prediction of stroke severity and outcome, and stratification of patients for stroke therapy in a practical and cost-effective manner.

## Methods

### Study population

105 consecutive AIS patients admitted to the local Stroke Unit of the Mediterranean Neurological Institute Neuromed – Institute of Research and Care, Molise, Italy were included prospectively in the study. Data collection consisted of a physical and neurological examinations and evaluations at the study clinic, urine test, blood draws. Data about biological determinations were collected from medical records. In particular, we evaluated glucose [25], albumin [26], TG, TC, LDL, HDL [27,28], INR, PTT, platelets [29], ESR [30], fibrinogen [31,32]. Fasting blood samples were collected at admission at Stroke Unit in approximately ten minutes. We assessed the participants for current smoking, antihypertensive medication, hypoglycemic medication, myocardial infarction history. The inclusion criteria for participation in the study was a diagnosis of acute ischemic stroke (ICD IX 434.91) admitted within the first 24 hours after symptom onset. Patients were excluded if they had a clinical record and documentation of the following: ischemic conditions including acute myocardial ischemia, peripheral vascular disease or shock, kidney failure, known inflammatory or malignant disease, transient ischemic attack, hemorrhagic stroke and finally a diagnosis other than stroke (e.g., migraine) or epileptic seizure. All respondents signed informed consent of the Mediterranean Neurological Institute Neuromed – Institute of Research which authorized the data treatment collected from all the patients during hospitalization.

### Stroke work-ups

All patients underwent brain MRI [1,5 T General Electric (GE), HDXt scanner] with diffusion-weighted imaging (DWI) that was reviewed by two neuroradiologists in AIS who were blinded to the clinical details and blood biomarkers determinated. The ischemic lesions have been considered according to size following the scheme: D1 – points (< 1.5 cm), D2 – nodular dimensions [1.5 - 3 cm] & parenchymal dimensions (> 3 cm), D3 – confluent dimensions, D4 – non confluent dimensions; and location anterior or posterior due to the diagnostic criteria [33]. In the case of multiple event the first event was used in the analysis. During the hospitalization, all patients with AIS had an extensive workup including transthoracic echocardiography carotid ultrasonography if required, computed tomography n = 10 (9.5%) or magnetic resonance angiography n = 91 (86.7%): CT or MRI angiography, and transthoracic saline contrast echocardiography. Blood pressure was measured twice in the supine position in the right upper arm using automated device [34,35], average of two measurements was used for analysis. Mean arterial pressure (MAP) was calculated as 1/3 systolic blood pressure (SBP) + 2/3 diastolic blood pressure (DBP). Hypertension was defined as DBP ≥ 90 mmHg and/or a SBP ≥ 140 mmHg or use of anti-hypertensive medication. Diabetes mellitus at admission was defined by one or two common criteria: high glucose serum levels or use of glucose lowering medication.

### Image analyses

**Figure 2:**
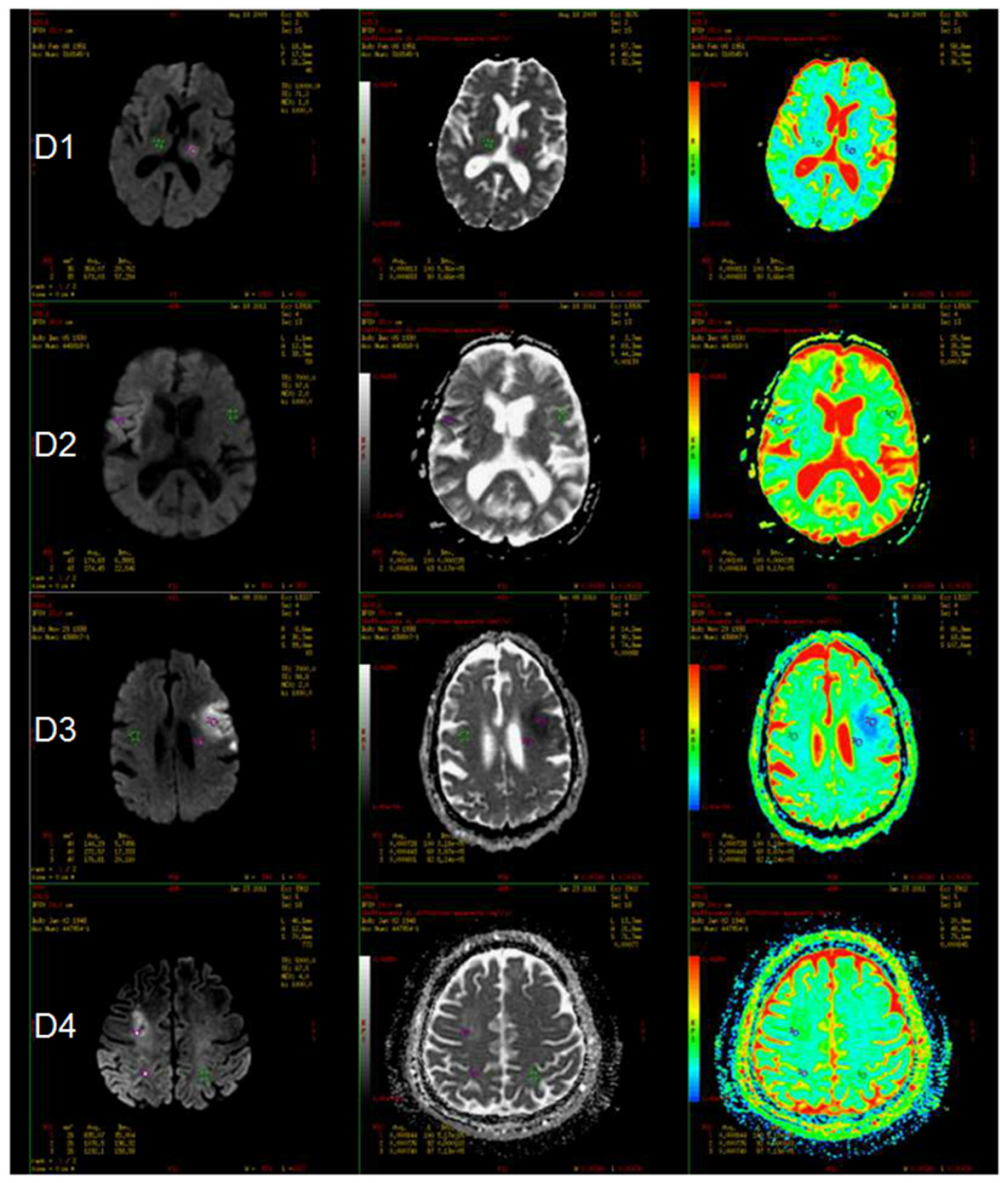
**Axial diffusion-weighted imaging (DWI) and apparent diffusion coefficient (ADC) maps in grey level and multi-chromatic that show acute ischemic lesion (D1 - D4): a lesion that is hyperintense on DWI and hypointense on ADC map is a characteristic magnetic resonance imaging (MRI) finding in acute ischemic infarction.** Note: regions of interest (ROI) positioned on normal parenchyma (1, green) and on acute ischemic areas (2,3, purple/red) in order to demonstrate hyperintensity on DWI corresponding to hypointensity on the ADC map in grey level. Multi-chromatic ADC maps show blue/light green areas in correspondence of ischemic lesions.

Patients were investigated at admission with a 1,5 T magnetic resonance scanner using a protocol including DWI [36] ( TR / TE: 7000 /98 msec, Matrix: 128 x 128, slice thickness / gap: 5.0 / 1.0 mm) and fluid attenuated inversion recovery imaging (TR / TE / TI: 8000 / 120 / 2000 msec, Matrix freq / phase: 288 x 192, slice thickness 5.0 / 1.0 mm ), to perform 20 to 23 slices. DWI was performed with 2 levels of diffusion sensitization (b.0 and 1000 sec / mm^2^). DWI lesion volumes were measured with MIPAV software (Medical Image Processing, Analysis and Visualization, version 3.0, National Institutes of Health, Bethesda, MD) and processed with Apparent Diffusion Coefficient (ADC) maps (Figure 2).

Two raters outlined regions of acute diffusion abnormality on the b.1000 image, consulting apparent diffusion coefficient and FLAIR sequences to distinguish acute from non-acute diffusion change. Acute diffusion lesions were defined on a slice-by-slice basis using a semiautomatic threshold approach by a rater blinded (BHB) to all clinical information [37]. Lesion volumes were calculated by multiplying slice thickness by the total lesion area.

### Biochemical assessment – laboratory analysis

Fasting blood samples were collected by venipuncture at admission to the Stroke Unit. Routine Dimension AX-R/Siemens, Stratus/Siemens, Adivia 120/Siemens, Coulter Maxm, Bct/Siemens were utilized for the determination of eleven plasma biomarkers values: glucose (mg / dL), albumin (g / dL), TG (mg / dL), TC (mg / dL), LDL (mg / dL), HDL (mg / dL), INR (ratio), PTT (seconds), platelets (value x 10.e^3^ / uL), ESR (mm), fibrinogen (mg / dL) routinely using standard assays [38]. All analyses were carried out at the same time by a biologist blinded to the diagnosis.

### NIHSS

Stroke severity was measured at the time of admission and after 7 days with the National Institutes of Health Stroke Scale (NIHSS). The NIHSS is a 15-item neurologic examination stroke scale used to provide a quantitative measure of stroke-related neurologic deficit by evaluating the effect of acute ischemic stroke on the levels of consciousness, language, neglect, visual-field loss, extra ocular movement, motor strength, ataxia, dysarthria, and sensory loss. Each item is scored with 3 to 5 grades, with 0 as normal and the final total score having a potential range of 0 to 42, with higher scores indicating greater stroke severity. Stroke severity measured at 7 days from admission by NIHSS was used to assess the independent association between variables (biomarkers) and outcome.

The mean NIHSS score evaluated at admission and at 7 days, all patients were divided to the one week evaluated NIHSS severity into groups with mild neurological deficits with NIHSS < 7; a group with severe neurological deficits with NIHSS ≥ 7. Finally, we divided the group with sever deficits in two subgroups: 7 ≤ NIHSS < 14, 14 ≤ NIHSS [39].

### Statistical analysis

Of the total sample, 62 patients (59.05%) were assessed with anterior stroke, 43 patients (40.95%) were assessed with posterior stroke: 9 patients ( 8.57% ) dead during the hospitalization, 6 patients ( 5.71% ) had repeated AIS event during their stay in the hospital.

The distribution of different lesions was as following: D1 lesions – 33 patients ( 31.43% ); D2 lesions – 18 patients ( 17.14% ) with nodular dimensions [1.5 – 3 cm] and 53 patients ( 50.48% ) with parenchymal dimensions (> 3 cm); D3 lesions – 23 patients ( 21.90% ); D4 lesions – 6 patients ( 5.71% ).

The mean NIHSS score (±SD) evaluated at 7 days was 6.47 ± 0.6. The distribution of different NIHSS severity groups at one week from admission was as following: NIHSS < 7 (n=70; 67.3%); NIHSS ≥ 7 (n=34; 32.7%); 7 ≤ NIHSS < 14 (n = 16; 15.4%); NIHSS ≥ 14 (n = 18, 17.3%).

χ^2^ test and t-test for independent samples to compare subgroups of patients were used. Multiple linear regression models were fitted to examine the independent correlation of biomarkers levels with cerebral lesion site and size, sex and age. For these analyses the biomarker levels were log transformed to yield normally distributed residuals. The normality of residuals was tested using a Shapiro - Wilk test.

Regression models assessing the blood markers quartiles difference from quartile one versus cerebral lesion size and sites were realized. The quartiles were used due to the large skew in the distribution resulting from individuals. Regression models assessing the blood markers quartiles versus cerebral lesion size and sites were fitted for all biomarkers outcomes and adjusted for age and sex. These findings were non dramatically affected by excluding antihypertensive medication, hypoglycemic medication from models, but by different values of efficacy, compliance, and dosage among treated individuals (data not shown). Each model significant P - value < 0.05 compared with the reference group of quartile one and relative Odds Ratios (OR) were found. The analyses were executed utilizing the SAS Statistical System 8.2.

## Competing interests

None of the authors had a personal or financial conflict of interest.

## Authors’ contribution

AK, GP, AC: study proposal, data collection, study design, multivariate statistical methods. CV, CC, AL, AP: study design and coordination, interpretation of results, writing the manuscript, critical revision. GG, VT, RG, MM: image analysis, collection and interpretation of data. MB, AMDeN: drafting a study codebook, critical draft revision. All authors read and approved the final manuscript.
